# Regulators of Tfh Cell Differentiation

**DOI:** 10.3389/fimmu.2016.00520

**Published:** 2016-11-23

**Authors:** Gajendra M. Jogdand, Suchitra Mohanty, Satish Devadas

**Affiliations:** ^1^T Cell and Immune Response, Infectious Disease Biology, Institute of Life Sciences, Bhubaneswar, India; ^2^Tumor Virology Lab, Infectious Disease Biology, Institute of Life Sciences, Bhubaneswar, India

**Keywords:** T follicular helper cell, CXCR5, ICOS, B cell lymphoma 6, programed death 1

## Abstract

The follicular helper T (Tfh) cells help is critical for activation of B cells, antibody class switching, and germinal center (GC) formation. The Tfh cells are characterized by the expression of CXC chemokine receptor 5 (CXCR5), ICOS, programed death 1 (PD-1), B cell lymphoma 6 (BCL-6), and IL-21. They are involved in clearing infections and are adversely linked with autoimmune diseases and also have a role in viral replication as well as clearance. On the one hand, Tfh cells are generated from naive CD4^+^ T cells with sequential steps involving cytokine signaling (IL-21, IL-6, IL-12, activin A), migration, and positioning in the GC by CXCR5, surface receptors (ICOS/ICOSL, signaling lymphocyte activation molecule-associated protein/signaling lymphocyte activation molecule) as well as transcription factor (BCL-6, c-Maf, and signal transducer and activator of transcription 3) signaling and repressor miR155. On the other hand, Tfh generation is negatively regulated at specific steps of Tfh generation by specific cytokine (IL-2, IL-7), surface receptor (PD-1, CTLA-4), transcription factors B lymphocyte maturation protein 1, signal transducer and activator of transcription 5, T-bet, KLF-2 signaling, and repressor miR 146a. Interestingly, miR-17–92 and FOXO1 act as a positive as well as a negative regulator of Tfh differentiation depending on the time of expression and disease specificity. Tfh cells are also generated from the conversion of other effector T cells as exemplified by Th1 cells converting into Tfh during viral infection. The mechanistic details of effector T cells conversion into Tfh are yet to be clear. To manipulate Tfh cells for therapeutic implication and or for effective vaccination strategies, it is important to know positive and negative regulators of Tfh generation. Hence, in this review, we have highlighted and interlinked molecular signaling from cytokines, surface receptors, transcription factors, ubiquitin ligase, and microRNA as positive and negative regulators for Tfh differentiation.

## Introduction

Follicular helper T cell (Tfh), a subset of helper CD4^+^ T cells, is involved in providing critical help for antibody maturation and germinal center (GC) formation (1). Robust regulation of Tfh cell response and subsequent antibody maturation are critical for infection clearance (2, 3), whereas aberrancy in controlling Tfh immune response is implicated in progression of autoimmune diseases such as systemic lupus erythematosus (SLE), arthritis, and type I/II diabetes (4–10). Moreover, Tfh cells were found to be choice of cells for HIV virus replication and survival (3, 11, 12). Because of association of Tfh cell with pathogenic as well as autoimmune diseases, attempts were made to increase or decrease Tfh cell number in order to reduce disease severity, pathology, and infection. As an example, improvement in beta cell function as a result of reduction in Tfh number was observed upon treatment of rituximab (anti-CD20) in patient with type I diabetes (13). In case of infection, HIV virus drives expansion of Tfh cell and blocking of programed death 1 (PD-1) receptor in HIV-infected humanized mice, inhibited HIV viral growth (14, 15). Thus, these studies prove that Tfh immune response can be regulated positively or negatively, and to better understand Tfh response modulation, it is important to characterize Tfh differentiation and its regulators.

Follicular helper T cells are characterized by expression of CXC chemokine receptor 5 (CXCR5) that is attracted toward CXCL-13 present in the B cell zone enabling these cells to enter interior of B cell follicle (16, 17). These cells are more precisely characterized by co-expression of ICOS (18), PD-1, cytokine IL-21 (19), and transcription factor B cell lymphoma 6 (BCL-6) (20–23) along with CXCR5. Moreover using knockout mice, it has been found that IL-6, IL-21, and BCL-6 are indispensable for Tfh differentiation (24). In contrast, Tfh differentiation in humans requires IL-12 and activin A signaling (25, 26). The CXCR5^+^ cells representing effector Tfh (27) or memory Tfh cells (1, 28–30) are also found in blood circulation. These circulating Tfh cells are functionally active to help B cells for antibody production (31). The Tfh cells can also be differentiated from other effector T cells (32) as exemplified in sustained viral infection where Th1 cells can be redirected to Tfh (33). Thus, Tfh cells can be generated from either differentiation of naive CD4^+^ T cells or from already differentiated effector T cells. In contrast, Tfh differentiation is negatively regulated by cytokine (IL-2, IL-7), surface receptor (CTLA-4, PD-1), transcription factors [B lymphocyte maturation protein 1 (Blimp-1), Klf-2], ubiquitin ligase, microRNA (miRNA), and Tfr cells. The mechanistic details for positive and negative regulation of Tfh differentiation are highlighted in this review.

## Tfh Differentiation and Inhibition

**Figure 1:**
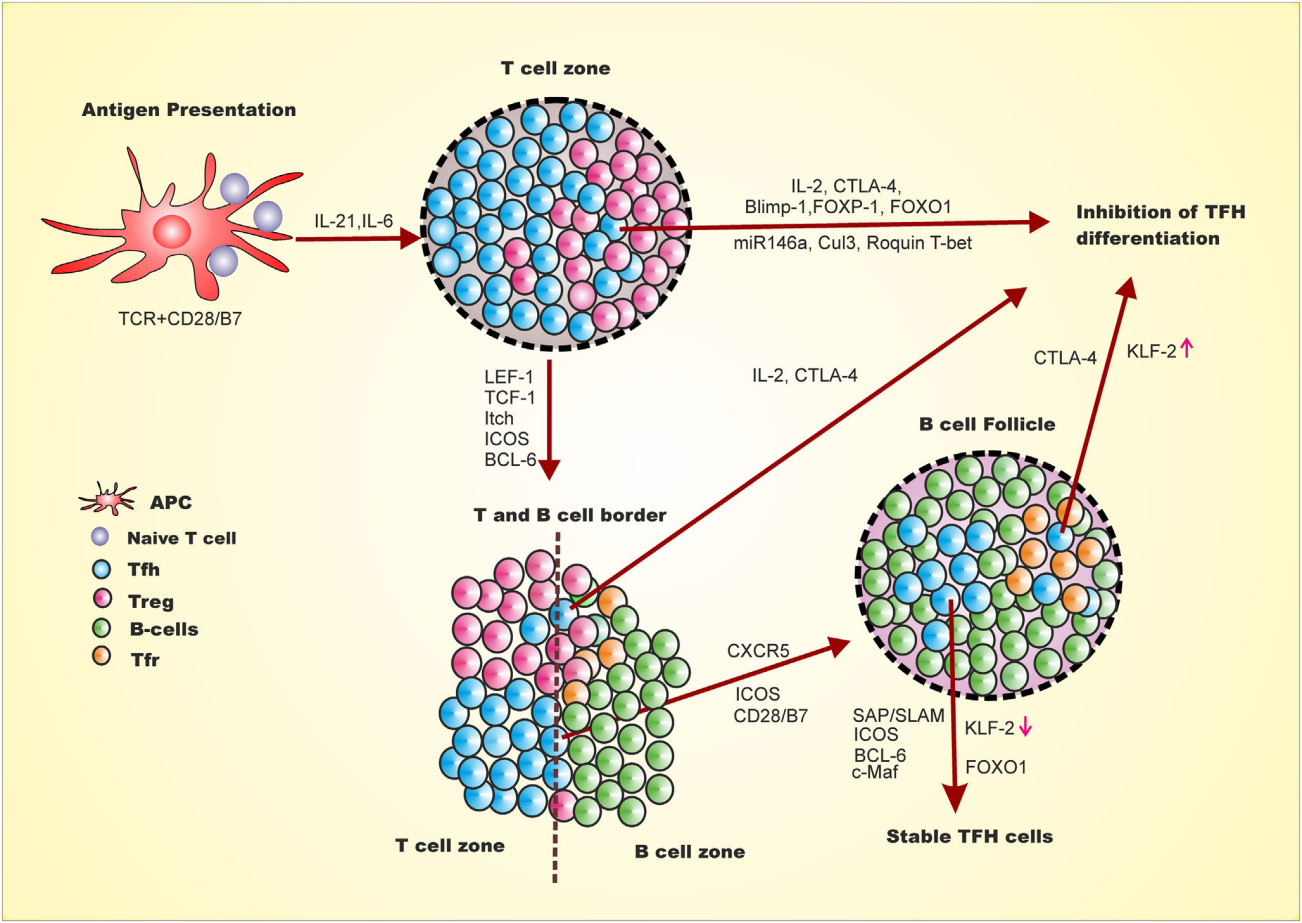
**Follicular helper T cell differentiation and inhibition is multi-step, multifactorial, spatiotemporal**. First step for naive CD4^+^ T cells to differentiate into Tfh involves antigen presentation by dendritic cells and CD28 co-stimulation leading to expression of LEF-1, TCF-1, ICOS and BCL-6. This early Tfh differentiation may be inhibited by expression of Blimp-1 FOCO-1, FOXp1, miR146a, Clul3, IL-2 and Roquin 1. The adjacent Treg cells either negatively regulate Tfh differentiation through CTLA-4 or promote the differentiation by reducing IL-2 at the vicinity of early Tfh cells. The expression of BCL-6 leads to the expression of CXCR5 and PD-1. The ICOS/ICOSL signaling downregulates KLF-2, which leads to upregulation of CXCR5 and downregulation of Blimp-1, T-bet, and CCR7. At this stage of Tfh differentiation, activin A also plays important role in downregulation of CCR7 and upregulation of CXCR5. CXCR5 upregulation leads to migration of these cells toward B cell follicle and interior of the germinal center. This step at T and B cell border is inhibited by IL-2 and Tfr through CTLA4. The migrated intermediate Tfh cells interacts with B cells through SAP/SLAM and signaling of which helps to stabilized Tfh generation which marks late event of Tfh differentiation and stable Tfh generation. Late stage of Tfh differentiation requires FOXO1 which at early stage is a negative regulator.

Positive and negative regulation of Tfh generation is multifactorial, multistep, and spatiotemporal (Figure 1) (24, 34–38). The expression of LEF-1, TCF-1 and later BCL-6 and ICOS on naive CD4^+^ T cells upon antigen presentation and co-stimulation, marks the early event of Tfh differentiation (39). This early event of Tfh differentiation is inhibited by Il-2, Blimp-1, FOXP1, FOXO1, Cul3, Roquin, and CTLA-4. Signaling from ICOS/ICOSL is involved in down-regulation of Blimp-1, T-bet, and CCR7 and upregulation of Tfh homing marker CXCR5 through regulation of *Klf2* (39, 40). In addition, activin A signaling is required for down-regulation of CCR7 and up-regulation of CXCR5 during Tfh differentiation from human naive CD4^+^ T cell (26). The down-regulation of CCR7 and up-regulation of CXCR5 leads to migration of early Tfh cells from T:B cell border to interior of B cell follicle. This stage of Tfh generation is inhibited by IL-2 and CTLA-4 from early Tfh, Treg, and Tfr (41, 42). Understanding how these early Tfh cells cross the barrier of intrinsic CTLA-4, Treg and Tfr regulation and/or generation of Tfh cells is spatiotemporal is yet to be discovered. Once this barrier is crossed, the late events in GC involve stable interaction of T and B cells through signaling lymphocyte activation molecule-associated protein (SAP)/signaling lymphocyte activation molecule (SLAM) signaling that further allows crosstalk between T and B cells. The SAP/SLAM signaling also regulates ICOS and CD40 expression. At this juncture, ICOS/ICOSL signaling is critical, as blocking ICOS signaling leads to reversion of these cells to other effector T cells by downregulation of CXCR5 and upregulation of CCR7 resulting in migration of these cells off the B cell follicle (39). At this particular point, Tfh differentiation can also be negatively regulated through IL-2, CTLA4 from Tfh or Tfr. Thus, cytokines, transcription factors, surface receptors, ubiquitin ligase, and miRNA act as positive and negative regulators of Tfh differentiation with mechanistic details as follows.

## Cytokine as Positive and Negative Regulators of Tfh Differentiation

Cytokine signaling is critical for cell survival, differentiation, proliferation, and also to undergo programed cell death. Along with antigen and costimulatory molecules, cytokine signaling plays a major role in driving naive CD4^+^ T cells to differentiate into specific effector T cell subsets. In studies with IL-21 and IL-6 knockout mice, it has been found that these cytokines are indispensable for Tfh differentiation. IL-21 cell intrinsically acts on the naive T cells to differentiate into Tfh through Vav1 (43), whereas IL-6 acts both intrinsically and extrinsically to enhance IL-21 production through c-Maf (44). Moreover IL-27, a heterodimeric cytokine is critical for the survival of activated cells as well as for the expression of Tfh marker. IL-27 enhances IL-21 production from naive CD4^+^ T cells and thereby supports GC formation and B cell functions (45). However in humans, along with IL-21 and IL-6, other cytokines such as IL-12 and TGF-β either solely or conjunctionally are involved in Tfh differentiation (25).

**Figure 2:**
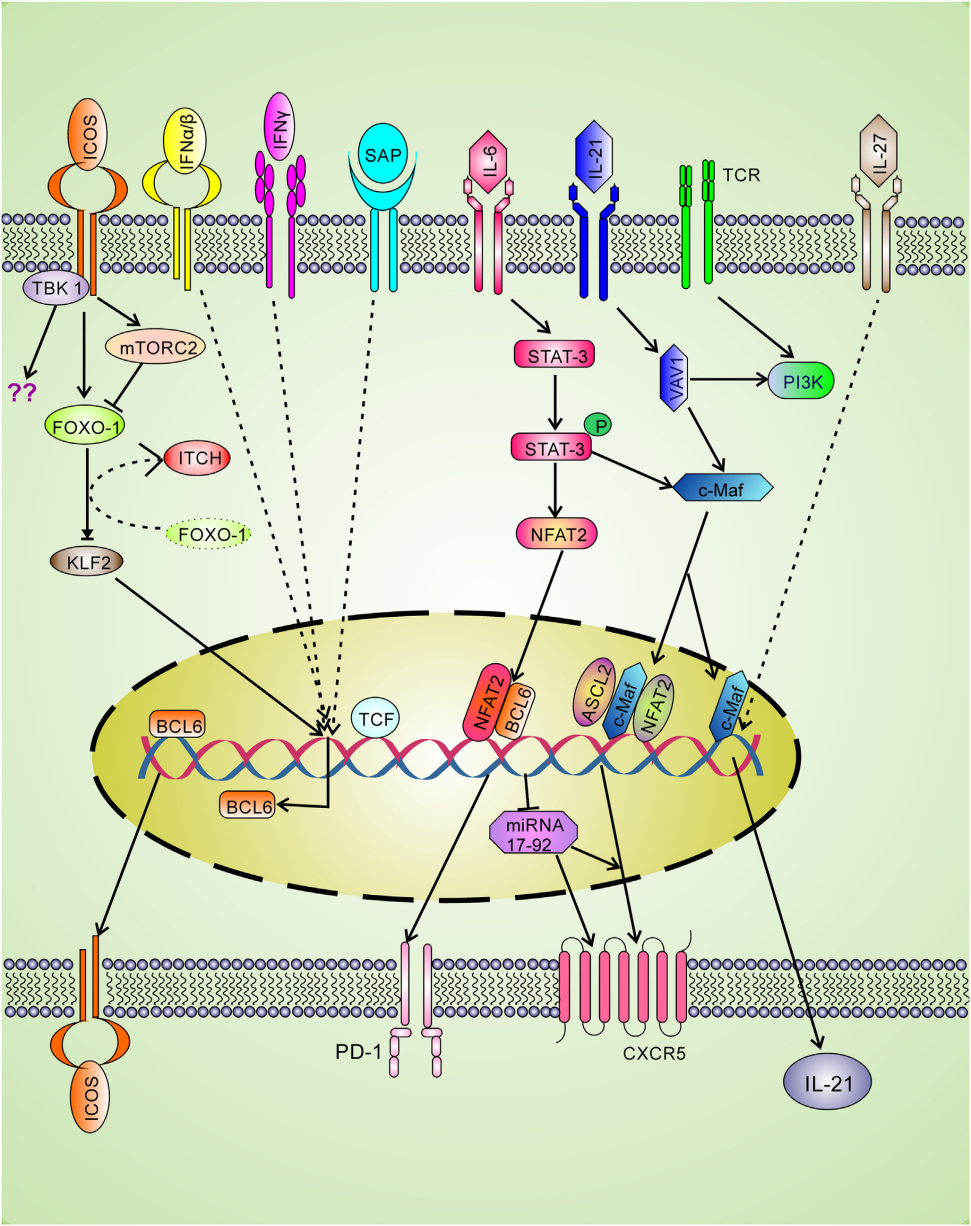
**Positive regulatory signaling for Tfh differentiation**. The signaling through T cell receptor (TCR) and co-stimulatory molecule leads to the expression of LEF-1 and TCF-1 which binds with *bcl-6 gene* and upregulates expression of the master transcription factor BCL-6. The BCL-6 expression leads to upregulate the expression of Tfh cell surface molecules CXCR5, ICOS, and PD-1. Signaling through ICOS is spatiotemporal and involved in BCL-6 and IL-21 production. ICOS through FOXO-1 down-regulates KLF-2, which in turn cannot block BCL-6 and CXCR5 expression. ICOS also induces mTORc2 which leads to degradation of FOXO-1. Ubiquitin ligase Itch also helps in FOXO-1 degradation. Strong TCR and ICOS signaling is required for the recruitment of TBK-1, a new ICOS downstream pathway for Tfh differentiation. L-21 signaling through Vav1 activates c-Maf that is involved in IL-21 production and CXCR5 expression. IL-27 can directly induce IL-21 production. The CXCR5 expression is also upregulated by ASCL2 and NFAT2 binding to *Cxcr5 gene*. BCL-6 positively regulates CXCR5 expression by repressing the repressor miRNA-17–92.

Cytokine interferons are involved in clearance of intracellular infection and seem to have positive roles in Tfh differentiation. The type I IFN-alpha/beta is involved in incomplete Tfh differentiation as they can induce BCL-6, CXCR5, and PD-1 expression through STAT1 signaling without IL-21 production (46). The type II interferon-gamma (IFN-γ) plays a positive role in Tfh differentiation as accumulation of Tfh was observed due to excess IFN-γ as shown in the Sanroque Lupus model (47). The blockade of IFN-γ reduces Tfh cells in Lupus demonstrating that IFN-γ plays an important positive role in Tfh generation. Mechanistically, excess IFN-γ signaling leads to increased BCL-6 expression (Figure 2) (47). In contrast, in late stage of Th1 differentiation, BCL-6 binds with the *Ifng* locus and represses over-production of IFN-γ (48). Therefore, it seems that BCL-6 and IFN-γ regulate each other by negative feedback mechanism.

The cytokines IL-2, IL-4, IL-7, IL-9, IL-15, and IL-21 signal through a common gamma chain receptor but have differential effect on T cell survival, proliferation, maintenance, and differentiation. Mechanistically, IL-2 positively regulates Th1 and Th2 differentiation through IL-12Rβ2 and IL-4ra expression, respectively, while it negatively regulates Th17 and Tfh differentiation by inhibiting the expression of IL-6 receptor α-chain (IL-6ra) (49). During influenza virus infection, impairment in GC and influenza-specific memory B cell formation was observed upon IL-2 administration. And this impairment was due to the suppression of Tfh cell differentiation by IL-2 (41). In another study in influenza infection, Treg cell decreased the availability of IL-2 to naive CD4^+^ T cells and thereby promoted Tfh differentiation and enhanced GC formation and antibody response. Moreover, depletion of Treg cells during the influenza infection leads to severe reduction in Tfh cells due to excess availability of IL-2 (50). A recent study depicts that IL-2 through activation of Akt and mTORc1 kinase drives cells toward Th1 rather than Tfh differentiation (51). The cytokine IL-7 acts a negative regulator of Tfh differentiation by activating signal transducer and activator of transcription 5 (STAT5) and thereby repressing *Bcl-6* and *Cxcr5* (52). Therefore, IL-21, IL-6, IL-27, IL-12, IFN-γ, and type I IFNs alpha/beta are positive regulators of Tfh differentiation, while IL-2 and IL-7 act as negative regulators of Tfh differentiation.

### Surface Receptors – Positive Regulator

#### CD28/B7 (CD-80 or CD86) Pathway

For naïve CD4^+^ T cell to differentiate into effector T cell, it requires antigenic peptide presentation through MCH-II and CD28 costimulation by antigen-presenting cells such as dendritic cells, macrophage, or B cells along with lineage driving cytokines. Signaling from CD28 by binding with its ligand CD80 (B7-1) or CD86 (B7-2) is critical for antibody class switching and GC formation (53, 54). Studies suggest that B7-1 and B7-2 have overlapping function as evidence from unaltered antibody response in mice lacks either of the molecules (53, 55). However, during *vaccinia* virus infection, expression of B7-2, but not B7-1, is critical for the generation of Tfh cells and thus plasma cell generation and virus specific antibodies (56). CD28 signaling positively regulates early stage of Tfh differentiation (expression of ICOS and BCL-6), and its signaling is also important at late stage of Tfh differentiation as exemplified in blocking B7 ligand during ongoing infection impaired Tfh response (39, 57, 58). Sustained signaling from CD28 is critical for Tfh survival as evidence from apoptosis of Tfh cells during influenza viral or *Citrobacter rodentium* infection into a mice strain that after initial priming selectively lose CD28 expression on CD4 T cells (58). Although CD28 signaling plays critical role in Tfh differentiation and maintenance, its downstream PI3k activation is dispensable for its generation and during infection with *Salmonella* in Roquin mutant mice, the generation Tfh does not depend on CD28 signaling (59). Thus, requirement of CD28 downstream signaling for Tfh generation may be infection specific, and still there is scope to determine unknown CD28 downstream signaling molecules.

#### CXC Chemokine Receptor 5

The presence of CXC chemokine receptor 5 (CXCR5), a hallmark Tfh cell surface marker, differentiates these cells from other T cell lineages (60, 61). Migration of T cells to the B cell zone is mediated by CXCR5, which is attracted toward CXCL13 ligand of B cell follicle (62), thereby promoting T:B cell contact. Although T cells are found in B cell follicle of immunized mice (selectively null for CXCR5 T cells), these mice were still found to have low GC number and decreased humoral immune response (63). Thus, CXCR5 is important for T cell positioning to follicular GC. However, it is also important that these cells should express low CCR7 expression as its signaling promotes retention of T cells to the T cell area of GC (64, 65). Signaling through activin A plays critical role in downregulation of CCR7 and upregulation of CXCR5 expression (26). Moreover, Tfh cells express high NFAT2 transcription factor that is involved in CXCR5 expression (66). Reduced CXCR5 expression was observed in NFAT2, NFAT1/2-deficient mice-infected LCMV Armstrong strain, indicating that nuclear factor of activated T cell (NFAT) plays critical role in Tfh differentiation by regulating CXCR5 expression (67).

#### ICOS/ICOSL Signaling

Upon antigen challenge and CD28 costimulation, ICOS is expressed on all effector T cells, and its signaling through ICOS-ICOSL is critical for Tfh generation and GC formation (18, 68). In patients with ICOS deficiency, the GC is severely damaged, and reduction in CXCR5^+^ CD45RO^+^ memory cells validates the importance of the ICOS in Tfh differentiation (69). During an acute viral infection, T cells that intermediately express IL-2 receptor α induces the expression of BCL-6 and CXCR5 through ICOS signaling leading to Tfh differentiation by 72 h of antigen challenge, without the help of B cell, whereas T cells expressing higher IL-2 receptor α induces Blimp-1 expression driving the cells toward other effector T cells subsets (70). After 72 h, the differentiated Tfh cells die due to the absence of ICOSL signal from B cells. Thus, sequential ICOS signaling is important for Tfh generation as well as maintenance.

Although PI3k is activated by signaling through T cell receptor (TCR), CD28, the ICOS-driven PI3k activation, is critical for Tfh cell as suggested by impaired Tfh generation in mice selectively defective in ICOS downstream signaling of the PI3k activation (71). Moreover, conditional deletion of catalytic subunit P110delta of PI3k also validate that the ICOS downstream PI3k pathway is important for Tfh generation (72). Other than known PI3K binding motif Tyr-x-x-Met (YxxM, where ‘x’ indicates any amino acid) of ICOS, Pedros et al. using SILAC (stable isotope labeling) approach showed that ICOS cytoplasmic tail has other two conserved motifs, one Proximal SSSVHDPNGE (IProx) and second AVNTAKK motif. Retroviral transduction of CD4^+^ T cells (TCR transgenic for LCMV peptide gp 61–80) with ICOS mutant in PI3k-binding site of IProx motif and adoptive transfer into ICOS^−/−^ mice and then LCMV infection fail to generate Tfh cells illustrating that IProx plays a critical positive role in Tfh differentiation (73). Importantly, IProx motif has homology with the conserved sequences with TRAF2 and TRAF3. Furthermore, IProx motif is involved in recruitment of serine threonine kinase TBK-1, and depletion of TBK-1 is associated with impaired Tfh generation. Strong TCR and ICOS signaling is needed for activation of TBK-1 pathway. Moreover, ICOS signaling also regulates IL-21 production through c-Maf, which in turn regulates Tfh differentiation (74). Recently, ICOS signaling has been associated with Tfh phenotype maintenance by FOXO1-mediated downregulation of transcription factor Kruppel-like factor 2 (KLF2) (39). KLF2 negatively regulates Tfh differentiation by directly binding to *Cxcr5, Cccr7, Psgl-1*, and *S1pr1* receptors, which leads to suppression of CXCR5 expression. In addition to Klf2, FOXO1 is also repressed by ICOS-induced mTORc2, thereby promoting Tfh differentiation (75). Although FOXO1 degradation is necessary for early-stage Tfh differentiation, however during late stage, it is required for transcription of several genes that help in Tfh differentiation (76).

#### Signaling Lymphocyte Activation Molecule-Associated Protein

The adaptor molecule SAP is very critical for the generation of antigen-specific long-lived plasma and memory B cells (77) by stabilizing T:B cell interaction (78). Two-photon imaging (79) showed that SAP deficiency selectively impairs T:B cell interaction rendering impaired T cell migration and retention in GC. SLAM/SAP signaling not only regulates stable T:B cell interaction but also exhibits a regulatory role in the expression of ICOS and CD40L (80). Thus, SLAM/SAP signaling positively regulates Tfh differentiation by stabilizing T and B cell interaction as well as the expression of ICOS and CD40L.

#### OX40/OX40L

Signaling through costimulatory molecule OX40 (CD138) and its ligand OX40L (CD252) is critical for T cell survival, proliferation, and differentiation (81–83). Classically, OX40 expression on T cells is considered to be transient with the exception during chronic LCMV clone 13 and *Plasmodium* infection wherein it is constantly expressed (84, 85). Enhancing signaling through OX40 is critical to control rodent malaria parasite infection by Tfh generation and GC response. Increasing OX40 signaling with OX40 agonistic antibody along with blocking PD-1 signaling leads to loss of Tfh generation by Blimp-1 expression and excessive IFN-γ production and thus impaired malaria parasite clearance (85). Therefore, it seems that the crosstalk between costimulatory and co-inhibitory receptor signaling is important for Tfh generation. In contrast, enhancing OX40 signaling during LCMV Cl 13 infection and initial priming of *Listeria monocytogenes* leads to inhibition of Tfh differentiation by increasing transcriptional repressor Blimp-1 (86, 87). Whereas following *L. monocytogenes* infection, enhancing OX40 signaling does not alter the Tfh number stating that the time of OX40 stimulation is an important factor for regulating Tfh differentiation. Moreover, infection with *L. monocytogenes* in mice deficient in OX40 and CD30 showed normal Tfh generation (86). However, OX40-deficient mice with LCMV Cl 13 infection showed impaired generation of Tfh cell, GC, and plasma cells (84). Thus, it seems that infection-specific tight regulation of OX40 signal is required for Tfh cell generation. Further, despite low memory CD4 T cell and low circulating memory B cells, intact antibody response to vaccination was observed in patient with OX40 deficiency (88). Aberrantly increased Tfh response leading to SLE is contributed by signaling through OX40 ligand (OX40L)–OX40 axis by augmentation of TCR signal *via* PI3K–Akt pathway (89). Thus, robust regulation of OX40/OX40L signaling is associated with positive and negative regulation of Tfh differentiation.

#### GITRL/GITR

During chronic LCMV infection and in collagen-induced arthritis (CIA) mice, the expression of glucocorticoid-induced TNF receptor family-related protein (GITR) was elevated on Tfh cells than non-Tfh cells (90, 91). Impaired Tfh generation and loss in control of chronic LCMV infection was observed in GITR^−/−^ mice. Moreover, administration of GITR-Fc protein greatly reduced CIA and joint damage by inhibiting Tfh generation and thereby autoantibody production (90). Mechanistically, GITR cell intrinsically expands Tfh number, thereby promoting antibody production to control chronic LCMV Cl13 infection (91). Thus, GITR signaling positively regulates Tfh generation.

### Surface Receptors – Negative Regulator

#### Programed Death 1

Programed death 1, a surface molecule expressed on exhausted T cells, is also expressed by Tfh cells. PD-1/PD-L1 and PDL-2 signaling is associated with the dephosphorylation of molecules downstream of TCR signaling. Mechanistically, PD-1 engagement inhibits ICOS signaling thereby regulating Tfh generation (92). However, IL-2 through STAT5 rescues inhibition of ICOS signaling by PD-1, and additionally, IL-2 itself through STAT5 negatively regulates Tfh differentiation. Therefore, it seems that many positive and negative regulatory pathways of Tfh differentiation are interlinked. The Tfr cells also express PD-1, regulate Tfh cells, and hence humoral immune response (93).

#### Cytotoxic T Lymphocyte Antigen 4

**Figure 3:**
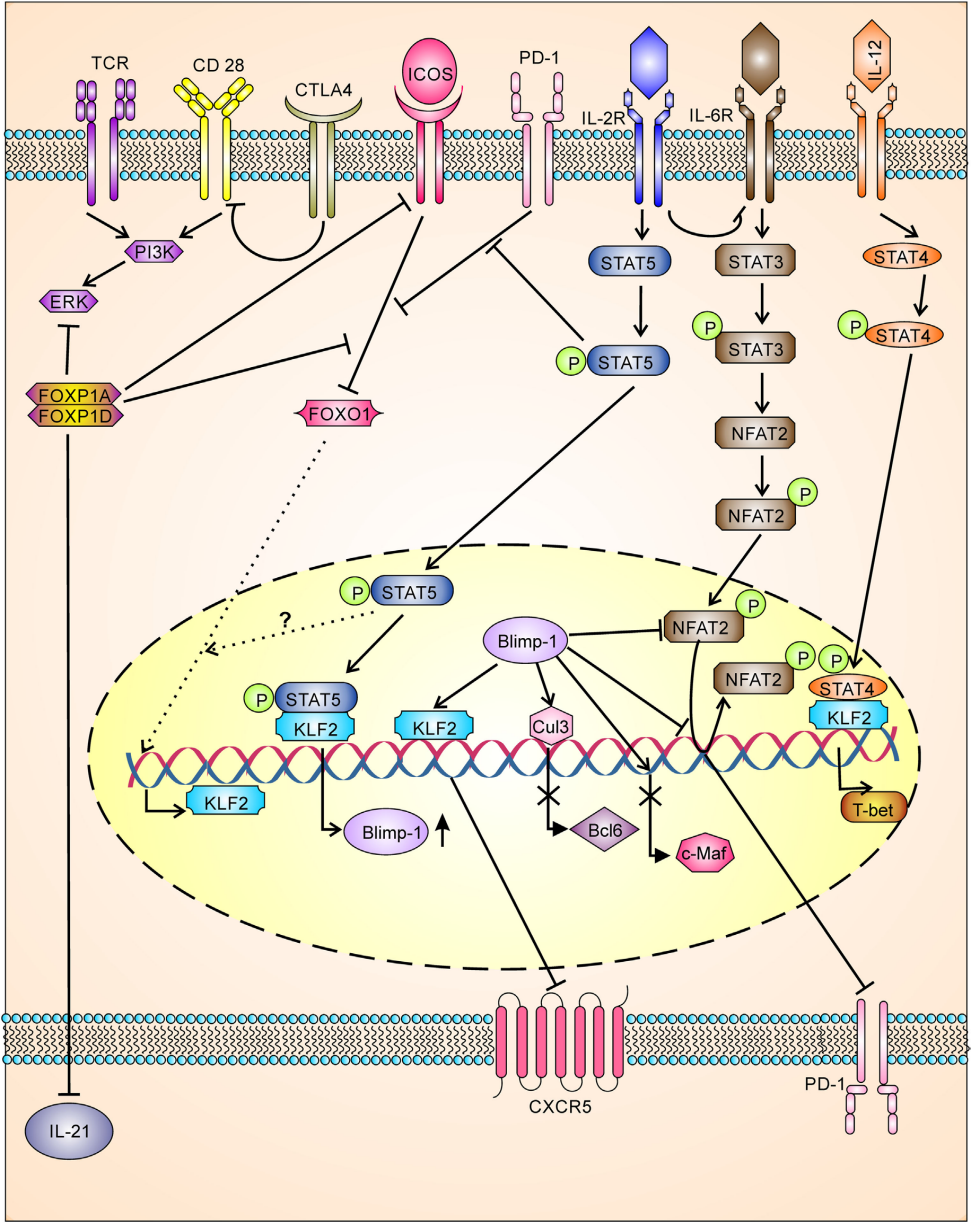
**Tfh differentiation is tightly negatively regulated having multiple checkpoints**. TCR and costimulatory signaling for Tfh differentiation is inhibited by CTLA-4 (by downregulation of B7-1 and B7-2) and FOXP1 (FOXP1A/FOXP1D). FOXP1 also inhibits ICOS receptor expression, ICOS/ICOS-L downstream signaling, and IL-21 production. Downstream signaling through ICOS/ICOS-L is also inhibited by PD-1 signaling. IOCS/ICOSL signaling inhibits FOXO-1, which in turn leads to upregulation of KLF2. KLF-2 inhibits CXCR-5 expression by binding to *Cxcr5* gene, whereas KLF-2 induces the production of Blimp-1 and T-bet. Signaling through IL-2/STAT5 inhibits PD-1-mediated inhibition of ICOS/ICOSL downstream signaling and enhances BLlimp-1 expression. Blimp-1, a transcriptional repressor, inhibits BCL-6, c-Maf, CXCR5, and PD-1. Blimp-1 regulates PD-1 expression directly inhibiting action of NFAT2 and or by displacing NFAT2 bound to *pdcd1* gene.

The cytotoxic T lymphocyte antigen 4 (CTLA-4), a regulatory T cell marker involved in regulation of effector T cells, was recently shown to be expressed by Tfh (42) and Tfr (42, 94) cells and negatively regulates Tfh differentiation. Downregulation or loss of CTLA-4 in Tfh cells and Treg cells resulted in augmented B cell response, while loss of CTLA-4 in Tfr was not able to augment B cell response. Mechanistically, Tfr and Treg control Tfh and humoral immunity by downregulating costimulatory molecule B7-1 and B7-2 through CTLA-4 (Figure 3) (42, 94). In contrast to this, earlier studies have shown that CTLA-4 activates ubiquitin ligase Itch resulting in inhibition of T cell activity (95), whereas recent studies have shown Itch has a positive regulator of Tfh differentiation (96). However, it is possible that CTLA-4 function may be controversial and *milieu* dependent or it may have new Tfh regulatory signaling pathways which are yet to be identified.

### Transcription Factors – As Positive and Negative Regulators of Tfh Differentiation

#### Signal Transducer and Activator of Transcription 3

Signal transducer and activator of transcription 3 (STAT3) plays a critical role as a positive regulator in Tfh differentiation (97). Mice with T cell-specific STAT3 deficiency showed reduced B cell response (98). Moreover, studies in human patients with STAT3 deficiency were reported with reduced circulating Tfh cells, defective IL-21 expression, and B helper activity supporting that STAT3 is a critical positive regulator of Tfh differentiation (40). Mechanistically, IL-6 enhances Tfh generation through expression of STAT3, which in turn regulates IL-21 production. In CD8 cells, IL-6-induced NFAT2/STAT3 was found to interact with *Pdcd1* gene promoter thereby regulating PD-1 (36). Thus, it is possible that IL-6-induced NFAT2/STAT3 might also be involved in the expression of PD-1 of Tfh cells.

#### B Cell Lymphoma 6

Follicular helper T cells cannot be generated in the absence of the transcriptional repressor BCL-6, implicating its importance in Tfh differentiation and persistence (99, 100). Ectopic BCL-6 expression leads to induction of CXCR5, ICOS, and PD-1 on CD4^+^ T cells (37, 101). BCL-6 promotes Tfh differentiation by inhibiting other T cell subset differentiation in a DNA binding-dependent manner. BCL-6 also regulates Th1 and Th17 cells by binding to T-bet and RORγt promoter thereby regulating IFN-γ and IL-17 production (101). Furthermore, BCL-6 directly binds with the AP-1 and blocks or subverts downstream TCR signaling of AP-1 and helps in Tfh generation (21). Yu et al. showed that the miRNAs, including miR-17–92 involved in CXCR5 repression, are repressed by BCL-6, driving the cells toward Tfh differentiation (101). BCL-6 has also been implicated in downregulation of P-selectin glycoprotein ligand 1 (PSGL1) simultaneously upregulating CXCR5 and PD-1 (102). Mechanistically, out of bric-a-brac, tramtrack, broad-complex (BTB) domain, a middle domain (also known as RDII), and a zinc finger domain of BCL-6, the BTB domain through interacting with BCOR (103), and middle domain RDII by associating with metastasis-associated protein 3 (MTA3) play critical role in Tfh differentiation (104, 105). Further acetylation of RDII domain at K379 prevents binding of MTA3, thereby inhibiting Tfh differentiation.

#### c-Maf

Cytokine IL-6 (44), IL-27 (106), and ICOS (74) induced c-Maf, which regulates IL-21 production, which in turn regulates Tfh generation. BCL-6 controls expression of most of the genes related to Tfh differentiation but does not have any role in IL-21 expression. Over-expression of c-Maf induces IL-21 production without signaling from IL-6/IL-4/STAT6 signaling (44). Mechanistically, c-Maf regulates IL-21 production through MARE site binding to the activated IL-21p and the CNS-2 enhancer (44). c-Maf induction is also involved in the expression of CXCR5 (107).

#### Aschaete Scute Homolog 2

Aschaete scute homolog 2 (Ascl2), a basic helix-loop-helix (bHLH) transcription factor, is specifically upregulated in Tfh cells. It positively regulates Tfh generation as blockade of Ascl2 signaling with Id3 leads to impaired Tfh generation (108). Ascl2 is involved in upregulation of the CXCR5 and downregulation of CCR7, thus helping in migration of these cells to the GC (108).

#### Interferon Regulatory Factor 4

Interferon regulatory factor 4 (IRF4) controls expression of BCL-6, as *Irf4*^−/−^ CD4^+^ T cells failed to express BCL-6, thus regulating the Tfh generation (109).

#### Nuclear Factor of Activated T Cell Proteins

The transcription factor NFAT consists of five members, namely, NFAT1 (NFATp or NFATc2), NFAT2 (NFATc or NFATc1), NFAT3 (NFATc4), NFAT4 (NFATx or NFATc3), and NFAT5. Signaling through Ca^2+^ regulates NFAT1, NFAT2, NFAT3, and NFAT4. The Ca^2+^ signaling-regulated NFAT1, NFAT2, and NFAT4 is critical for T cell activation, differentiation, and maintenance of tolerance (110–112). Recently, it has been shown that Tfh cells express high NFAT2 transcription factor that is involved in CXCR5 expression. Reduced expression of Tfh markers CXCR5, ICOS, and PD-1 was observed in NFAT2, NFAT1, 2-deficient mice infected with LCMV Armstrong strain, which illustrates important positive role for NFAT1 and NFAT2 in Tfh differentiation (67). In contrast, unexpectedly, augmented GC reaction was observed upon immunization in mice deficient in NFAT2. However, in these NFAT2-deficient mice, only Tfr population was hampered, indicating that NFAT2 intrinsically downregulates CXCR5 in Tfr than Tfh (66).

#### Signal Transducer and Activator of Transcription 5

During T cell activation, continuous STAT5 signaling inhibits Tfh generation by regulating expression of CXCR5, c-Maf (113), and BCL-6 (113, 114). Therefore, *in vivo* inhibition of STAT5 signaling leads to enhanced Tfh cell and GC formation. In the absence of Blimp-1, STAT 5 failed to inhibit Tfh cell formation, whereas over-expression of Blimp-1 in STAT5-deficient T cells resulted in Tfh differentiation. Thus, IL-2/STAT5 axis negatively regulates Tfh differentiation by regulating Blimp-1 (113).

#### B Lymphocyte Maturation Protein 1

B lymphocyte maturation protein 1, a transcriptional repressor, plays a critical role in the regulation of terminal B (115) as well as T cell differentiation, maturation, survival, and homeostasis (116, 117). It negatively regulates Tfh differentiation by opposing the action of BCL-6 (100) by binding to multiple loci of *Bcl-6* genes (118). Blimp-1 also inhibits PD-1 expression by directly repressing NFAT2 (a positive regulator of PD-1), or by binding to *PD-1* locus changing the chromatin structure, thereby resulting in the dissociation of NFAT2 from its site (78). Thus, Blimp-1 negatively regulates Tfh differentiation by downregulating BCL-6 and PD-1.

#### T-Bet

The master transcription factor T-bet and BCL-6 for Th1 and Tfh cells, respectively, have been shown to be co-expressed during early and late stages of differentiation and regulate each other to drive the cells either toward Th1 or Tfh type (119). In the early stages of Th1 cell differentiation, IL-12 priming through STAT4 induces IL-21 and BCL-6, but during late phase of differentiation, STAT4-induced T-bet represses BCL-6 leading naive CD4^+^ T cell to differentiate into Th1. Recent finding has shown that during late event of Tfh cell differentiation, low expression of KLF-2 at early stages leads to BCL-6 expression and high expression of KLF-2 during later phase results in downregulation of BCL-6 by upregulating BCL-6 antagonistic Blimp-1 and high expression of T-bet (40). Thus, KLF2 may regulate T-bet and thereby negatively regulate Tfh differentiation, but still our knowledge is unclear for molecules that are involved during late event of Th1 differentiation that negatively regulates Tfh differentiating molecules.

#### E Protein and Its Natural Repressors Id2 and Id3

E protein and its natural repressors, the inhibitor of DNA-binding protein (Id1–Id4), play a critical role in Tfh differentiation. The generation of Tfh cells was increased upon depletion of Id2 *via* RNA-mediated interference. Moreover, over-expression of Id3 inhibits Tfh generation during LCMV infection or following immunization with protein (120). Ectopic expression of E protein E47 enhanced CXCR5 expression, while Id2 inhibits CXCR5 expression. Chromatin immunoprecipitation (ChIP) followed by deep sequencing has revealed that Bcl-6 binds with Id2 locus repressing Id2 expression thereby positively regulating Tfh differentiation (120).

#### Forkhead/Winged Helix Transcription Factor

##### FOXP1

FOXP1 intrinsically negatively regulates naive CD4^+^ T cell differentiation into effector function by antagonizing FOXO1 and also through inhibition of MEK and ERK signaling (121). FOXP1-deficient naive CD4^+^ T cells upon TCR stimulation differentiate preferentially into Tfh type, indicating its critical negative role in Tfh differentiation (122). FOXP1, through its isoforms FOXP1A and FOXP1D, directly suppresses IL-21 production, ICOS expression, and ICOS downstream signaling (122).

##### FOXO1

FOXO1, a member of forkhead box transcription factor family, is involved in T cell homeostasis and nutrients and growth factor adaptation. It controls lymphocyte trafficking by regulating the adhesion molecule L-selectin, chemokine receptor CCR7, and transcription factor KLF2 (123–125). FOXO1 deficiency led to enhanced expression of KLF 2 that negatively regulates Tfh generation. In contrast, viral infection studies show that degradation of FOXO1 by ubiquitin E3 ligase Itch enhances Tfh differentiation (96). Moreover, a recent study showed that during late stage of Tfh differentiation, FOXO1 can positively regulate Tfh differentiation, although its regulatory molecules are yet to ascertain (76). Therefore, FOXO1 regulates Tfh differentiation positively as well as negatively that may be time dependent involving unknown molecules.

#### Kruppel-Like Factor 2, Also Known as Lung Kruppel-Like Factor

Kruppel-like factor 2, member of zinc finger transcription factor, is involved in T cell homeostasis, trafficking, and survival, and KLF2-deficient mice showed reduction in peripheral T cells (126–128). Lee et al. showed that the KLF2 deficiency or low levels of KLF2 have been shown to be associated with enhanced Tfh cell differentiation. Moreover, over-expression of KLF2 leads to blocking or reduction of Tfh cell differentiation and thereby resulting in reduced GC formation (40). Mechanistically, KLF2 negatively regulates Tfh differentiation by directly binding to *Cxcr5, Ccr7, Psgl-1*, and *S1pr1* and thus negatively regulating homing receptor (39, 40). The other mechanism by which KLF2 regulates Tfh differentiation is through the induction of Blimp-1, T-bet, and GATA3 expression (40).

#### LEF-1 and TCF-1

The transcription factors LEF-1 and TCF-1 act as a positive regulator of Tfh differentiation and expressed before the expression of Tfh marker CXCR5. During early stage of viral infection, virus-specific Tfh cells reciprocally express TCF-1 and Blimp-1 (129). The TCF-1 deficiency led to poor differentiation of Tfh cells and their maintenance. Mechanistically, TCF-1 regulates Tfh differentiation by directly binding to the *Bcl6* promoter and *prdm 5*′ regulatory region enhancing BCL-6 and repression of Blimp-1 protein (130). Moreover, LEF-1, by enhancing IL-6 receptor and ICOS expression, promotes Tfh differentiation (131).

## Ubiquitin E3 Ligase

### Ubiquitin E3 Ligase Itch as Positive Regulator of Tfh Differentiation

Recent studies have shown that during acute viral infection, ubiquitin ligase Itch plays an important role in Tfh differentiation, GC formation, and humoral immune response (96). The action of Itch was found to be intrinsic and upstream of BCL-6 as forced BCL-6 expression in *Itch*^−/−^ mice restored Tfh differentiation. Mechanistically, Itch activates FOXO1 ubiquitination and degradation, thereby helping Tfh differentiation. The deletion of FOXO1 in Itch^−/−^ mice rectifies the Tfh differentiation validating the fact that Itch mediated degradation is crucial for Tfh differentiation (96).

### Ubiquitin E3 Ligase Cullin3 as Negative Regulator of Tfh Differentiation

The E3 ligase Cullin3 (Cul3) binds to BCL-6 and negatively regulates Tfh differentiation by ubiquitination of histone proteins. Knocking down E3 ligase Cul3 from CD4 cells resulted in over-expression of basic leucin zipper transcription factor, ATF-like (Baft), a target of BCL-6 leading to exaggerated or enhanced Tfh generation during antigen challenge (132).

### Ubiquitin E3 Ligase ROQUIN as Regulator of Tfh Differentiation

Roquin 1 (Rc3h1) and Roquin 2 (Rc3h2) are paralogous proteins made up of really interesting new gene (RING) finger, ROQ domain, and zinc finger domain and regulates Tfh differentiation at posttranscriptional level. In Roquin^san^ mice, substitution of M199R within ROQ domain of Roquin 1 leads to enhanced expression of ICOS, thereby promoting aberrant Tfh generation causing lupus like autoimmunity (133). Roquin 1 mechanistically regulates Tfh differentiation by repressing mRNA of ICOS and Ox40 expression (134, 135). Unlike Roquin^san^ mice, Roquin null mice do not show increase in the ICOS expression but fail to generate aberrant Tfh cells, suggesting that there is involvement of other compensatory regulatory molecule (136). Roquin 2 negatively regulates Tfh differentiation in the absence of Roquin 1 (134, 135). In contrast, hampered antigen-specific Tfh response was observed in mice with T cells specific deletion of Roquin RING domain depicting Roquin RING domain positively regulates Tfh differentiation. Mechanistically, Roquin directly binds with AMPKα subunit and thus represses adenosine monophosphate-activated protein kinase (AMPK) activity (137).

## microRNA

microRNA regulates gene expression at posttranscriptional level and thereby is crucially involved in regulation proliferation, differentiation, and programed cell death. Studies depict critical positive and negative roles of microRNA in the Tfh differentiation. In 2009, Yu et al. showed that the microRNAs, including miR-17–92 involved in CXCR5 repression, are repressed by BCL-6-driving cells toward Tfh differentiation (101), while during viral infection, over-expression of miR-17–92 is importantly involved in Tfh differentiation (138, 139). Thus, miR-17–92 may regulate Tfh differentiation positively or negatively. During chronic low-grade inflammation, miR-155 is involved in progression of inflammatory disease by increasing the number of Tfh cell number. On the other hand, miR-146a counterregulates microRNA-155 and reduces the number of Tfh cells (140). Mechanistically, miR-146a regulates ICOS/ICOSL signaling by repressing messenger RNA involved in ICOS expression (141). Thus, micorRNA regulates Tfh differentiation positively as well as negatively.

## Conclusion

Follicular helper T cells are known for their critical help in GC formation, and B cell maturation for infection clearance is also implicated in the autoimmune diseases and virus replication. In order to clear infection and to prevent immunopathology, precise manipulation of Tfh regulatory molecular network is needed to generate adequate Tfh cell number. Adequate Tfh cell number may be generated by manipulating Tfh regulatory molecules such as cytokines, transcription factors, surface receptors, ubiquitin ligase, and miRNAs. Further research is needed to ascertain regulatory molecules either positive or negative for generation of Tfh from effector T cells. The generation of adequate Tfh number and its critical help for optimum GC formation and B cell maturation will be helpful for effective vaccination strategy.

## Author Contributions

GJ and SD discussed and revived the literature; GJ drafted the manuscript. GJ, SD, and SM prepared illustrations, discussed, and revived the manuscript. All the authors critically revised the manuscript for intellectual content and approved it for publication.

## Conflict of Interest Statement

The authors declare that the research conducted has no commercial or financial involvement that could be considered as potential conflict of interest.
